# Loss of the ETR1 ethylene receptor reduces the inhibitory effect of far-red light and darkness on seed germination of *Arabidopsis thaliana*

**DOI:** 10.3389/fpls.2014.00433

**Published:** 2014-08-28

**Authors:** Rebecca L. Wilson, Arkadipta Bakshi, Brad M. Binder

**Affiliations:** Department of Biochemistry, Cellular, and Molecular Biology, University of TennesseeKnoxville, TN, USA

**Keywords:** ethylene receptors, ethylene, phytochrome, light signaling, cross-talk, seed germination

## Abstract

When exposed to far-red light followed by darkness, wild-type *Arabidopsis thaliana* seeds fail to germinate or germinate very poorly. We have previously shown that the ethylene receptor ETR1 (ETHYLENE RESPONSE1) inhibits and ETR2 stimulates seed germination of *Arabidopsis* during salt stress. This function of ETR1 requires the full-length receptor. These roles are independent of ethylene levels and sensitivity and are mainly mediated by a change in abscisic acid (ABA) sensitivity. In the current study we find that *etr1-6* and* etr1-7* loss-of-function mutant seeds germinate better than wild-type seeds after illumination with far-red light or when germinated in the dark indicating an inhibitory role for ETR1. Surprisingly, this function of ETR1 does not require the receiver domain. No differences between these mutants and wild-type are seen when germination proceeds after treatment with white, blue, green, or red light. Loss of any of the other four ethylene receptor isoforms has no measurable effect on germination after far-red light treatment. An analysis of the transcript abundance for genes encoding ABA and gibberellic acid (GA) metabolic enzymes indicates that *etr1-6* mutants may produce more GA and less ABA than wild-type seeds after illumination with far-red light which correlates with the better germination of the mutants. Epistasis analysis suggests that *ETR1* may genetically interact with the phytochromes (phy), *PHYA* and *PHYB* to control germination and growth. This study shows that of the five ethylene receptor isoforms in *Arabidopsis*, ETR1 has a unique role in modulating the effects of red and far-red light on plant growth and development.

## INTRODUCTION

Ethylene is a gaseous plant hormone that is involved in many developmental and physiological processes in higher plants (Mattoo and Suttle, 1991; Abeles et al., 1992). In *Arabidopsis thaliana*, there are five receptor isoforms that mediate responses to ethylene called ETHYLENE RESPONSE1 (ETR1), ETR2, ETHYLENE INSENSITIVE4 (EIN4), ETHYLENE RESPONSE SENSOR1 (ERS1), and ERS2 (Chang et al., 1993; Hua and Meyerowitz, 1998; Hua et al., 1998; Sakai et al., 1998). Genetic studies have shown that in the absence of ethylene, the receptors positively regulate CONSTITUTIVE TRIPLE RESPONSE1 (CTR1) which acts as a negative regulator of the pathway. The role of CTR1 is to inhibit downstream components of the pathway and prevent ethylene responses (Kieber et al., 1993). According to current models, ethylene binding to the receptors reduces the activity of the receptors. This leads to reduced activity of the CTR1 kinase resulting in reduced phosphorylation of EIN2 protein (Chen et al., 2011; Ju et al., 2012; Qiao et al., 2012). This reduction in EIN2 phosphorylation leads to a decrease in ubiquitination of EIN2 causing a rise in EIN2 protein levels and proteolytic separation of the C-terminal portion of the protein from the N-terminal portion (Qiao et al., 2009, 2012; Ju et al., 2012; Wen et al., 2012). The C-terminal region of EIN2 causes a rise in the levels of the EIN3 and EIL1 transcription factors (Guo and Ecker, 2003; Yanagisawa et al., 2003; Gagne et al., 2004) which leads to most ethylene responses.

The plant ethylene receptors fall into two subfamilies with subfamily 1 comprising ETR1 and ERS1 and subfamily 2 comprising ETR2, EIN4, and ERS2. These receptors have homology to bacterial two-component receptors that signal via histidine autophosphorylation followed by phosphotransfer to an aspartate on a receiver domain. However, only subfamily 1 receptors have histidine kinase activity *in vitro* with the subfamily 2 receptors having serine/threonine kinase activity *in vitro* (Gamble et al., 1998; Moussatche and Klee, 2004). ETR1 is unique in that it is the only ethylene receptor in *Arabidopsis* with both histidine kinase activity and a receiver domain and it may be the only receptor with histidine kinase activity *in vivo* (Moussatche and Klee, 2004). ETR1 histidine kinase activity is not required for ethylene signaling in plants (Wang et al., 2003; Xie et al., 2006; Qu et al., 2007) but does modulate signal output via an unknown mechanism (Binder et al., 2004; Kim et al., 2011; Hall et al., 2012). All of the *Arabidopsis* ethylene receptor isoforms are involved in ethylene signaling (Hua et al., 1995, 1998; Hall and Bleecker, 2003). However, even though the receptors have overlapping roles for certain traits, it is also clear that they have non-redundant roles (Hall and Bleecker, 2003; Binder et al., 2004, 2006; Seifert et al., 2004; O’Malley et al., 2005; Xie et al., 2006; Qu et al., 2007; Plett et al., 2009a,b; Liu et al., 2010; Kim et al., 2011). In some cases, individual isoforms have roles that are opposite from the other isoforms in the control of certain phenotypes (Binder et al., 2006; Liu et al., 2010; Wilson et al., 2014). This is likely to be a common attribute of ethylene signaling in land plants since certain receptors have a prominent role in controlling specific phenotypes in other species including both eudicots and monocots (Tieman et al., 2000; Kevany et al., 2007; Chen et al., 2009; Wuriyanghan et al., 2009). The mechanistic basis for these unique roles is unknown.

Seed germination is a critical developmental stage of the plant that is controlled by both hormones such as abscisic acid (ABA), gibberellic acid (GA), and ethylene and environmental factors such as light quality and quantity (reviewed by Bentsink and Koorneef, 2008; Linkies and Leubner-Metzger, 2012). Ethylene stimulates germination of *Arabidopsis* seeds (Bleecker et al., 1988; Wilson et al., 2014) and ethylene insensitive *etr1-1* mutants have diminished germination (Chiwocha et al., 2005). Ethylene appears to function in this process by antagonizing the inhibitory effects of ABA on germination (Chiwocha et al., 2005; Linkies et al., 2009). However, we recently showed that loss of ETR1 enhances and loss of ETR2 diminishes seed germination during salt stress (Wilson et al., 2014). These contrasting roles for ETR1 and ETR2 are not predicted by current models of ethylene signaling and appear to largely be independent of ethylene. Rather, ETR1 and ETR2 are affecting seed germination during salt stress predominantly via regulation of ABA synthesis or signaling (Wilson et al., 2014). These prior observations suggested that there might be other traits regulated by the ethylene receptors that are unpredicted for by current models of ethylene signaling.

Light intensity and quality affect *Arabidopsis* seed germination and the phytochrome (phy) family of photoreceptors has a critical role in this regulation (Shinomura et al., 1994, 1996). The phy are evolutionarily related to the ethylene receptors with both receptors having homology to bacterial two-component receptors and are thought to have been acquired from cyanobacteria (Bleecker, 1999; Mount and Chang, 2002). It is noteworthy that even though the phy are evolutionarily related to the ethylene receptors and also contain putative histidine kinase domains, plant phy have serine/threonine protein kinase activity (Yeh and Lagarias, 1998; Fankhauser et al., 1999) much like the subfamily two ethylene receptors. The phy are red/far-red photoreversible receptors that affect many developmental and growth processes in plants including germination (Neff et al., 2000). Illumination with red light drives phy to the active form of the receptor that is far-red absorbing (P_FR_) and illumination with far-red light drives it toward the red absorbing form (P_R_) that is inactive. There are complex interactions between light and phytohormones in the control of germination (reviewed by Bae and Choi, 2008; Finkelstein et al., 2008; Jaillais and Chory, 2010; Lau and Deng, 2010; Leivar and Quail, 2010). Here we report that loss of ETR1 reduces the effect of far-red light on germination and is affecting the transcript abundance of genes encoding metabolic enzymes for both GA and ABA. Epistasis analysis shows that *ETR1* genetically interacts with *PHYA* and *PHYB*. However, the effects of ETR1 on seed germination may be independent of light suggesting a model where ETR1 acts in parallel with the photoreceptors to control seed germination.

## MATERIALS AND METHODS

### PLANT MATERIALS AND CHEMICALS

The *etr1-6*, *etr1-7*, *etr2-3*, and *ein4-4* mutants are lab stocks that have been previously characterized (Hua and Meyerowitz, 1998). The *phya-t* (SALK 014575C) and *phyb-9* (CS6217) mutants were obtained from the *Arabidopsis* Biological Resource Center and have previously been described (Rösler et al., 2007; Sung et al., 2007). The receptor combinatorial mutants and transformants used in this study have previously been described (Hua and Meyerowitz, 1998; Kim et al., 2011; Wilson et al., 2014). All mutants are in the Columbia background. GA was from ACROS Organics (Belgium) and the ABA biosynthesis inhibitor norflurazon was from Fluka (Switzerland). L-α-(2-amino ethoxyvinyl)-glycine (AVG) was a gift from Rohm Haas (Philadelphia).

The *etr1-6;phya-t* and *etr1-6;phyb-9* mutants were obtained by crossing *phya-t* to *etr1-6* and *phyb-9* to *etr1-6*. The double mutants were selected for in the F2 generation by first growing seedlings in constant far-red light to identify homozygous *phya-t* mutants or constant red light to identify homozygous *phyb-9* mutants. Tall seedlings were transferred to soil and subsequently genotyped with dCAPs primers to identify homozygous *etr1-6* plants. The dCAPs primers used for genotyping *etr1-6* were 5′-ACTCGTTGAAGTCGTCCCTGATC-3′ (forward) and 5′-ATGTGAGAGAGCTACAGCCAC-3′ (reverse). These primers yield an expected polymerase chain reaction (PCR) product of 121 bp. Digestion of this PCR product with BslI results in a 23 and 98 bp fragment for wild-type *ETR1* and does not digest product from *etr1-6*, while digestion with Hpy188I results in a 24 and 97 bp product from *etr1-6* and does not digest PCR product from wild-type *ETR1*. Homozygous F3 or F4 seeds were used for germination assays and analysis of hypocotyl lengths.

### SEED GERMINATION EXPERIMENTS

To minimize biological variation, seeds were harvested on the same day from plants grown together under similar conditions (Hensel et al., 1993), stored in a desiccator at room temperature, and allowed to after-ripen for at least 3 weeks. These were then mechanically sorted as previously described (Ellwell et al., 2011). Seeds between 250 and 300 μm in size were surface sterilized in 70% ethanol for 30 s, dried on filter paper and placed on agar plates containing half-strength Murashige and Skoog (MS) basal medium with Gamborg’s vitamins (Sigma, St. Louis, MO, USA), pH 5.7, 0.8% (w/v) agar, with no added sugar. Plates were sealed with micropore surgical tape (3M, St. Paul, MN, USA) so that ethylene did not accumulate (Buer et al., 2003). Twenty seeds of one genotype were plated per plate with a spacing of 5 mm between seeds and three plates per genotype per condition were used in each experiment.

Where used, GA and the ABA synthesis inhibitor, norflurazon were prepared as 10,000× stocks in ethanol, filter sterilized, and added to the media at the indicated concentration after autoclaving the media. Solvent control plates contained 0.01% ethanol. In some cases, 5 μM AVG was added to block ethylene biosynthesis. Ethylene treatments were conducted by placing the plates of seeds in sealed containers and injecting ethylene to yield the indicated concentrations.

Unless otherwise specified, seeds were treated with 45–55 μmol m^-2^ s^-1^ white light for 4 h. This was followed by a 3 h treatment with 12 μmol m^-2^ s^-1^ blue (λ_max_ = 470 nm), green (λ_max_ = 525 nm), red (λ_max_ = 672 nm), or far-red (λ_max_ = 732 nm) light. Monochromatic light was provided by LED arrays (Quantum Devices, Inc., Barneveld, WI, USA). For positive controls, seeds were treated for 3 h with 45–55 μmol m^-2^ s^-1^ white light. After light treatment, seeds were allowed to germinate for 7 days in darkness at which time germination was evaluated. For dark controls, seeds were treated for 5 min with far-red light immediately after sowing without a pre-treatment with white light (Oh et al., 2007) and then allowed to germinate in darkness for 7 days. In some experiments, seeds were germinated on 150 mM NaCl under continuous white light.

Germination was scored as the visible rupture of the testa (seed coat). After each experiment, plates containing seeds that did not germinate were transferred to white light conditions (16 h of light/8 h of dark photoperiod) and germination evaluated after 7 days. In all cases, seeds reached at least 95% germination showing that the seeds were viable.

### ETHYLENE MEASUREMENTS

To measure ethylene production, 22 mg of seeds were placed on 1 mL of half-strength MS media in sealed 6 mL glass vials, treated with white or far-red light, and ethylene levels in the headspace measured every 6 h over several days using an ETD-300 photoacoustic ethylene detector (Sensor Sense, The Netherlands) as previously described (Wilson et al., 2014).

### HYPOCOTYL GROWTH EXPERIMENTS

To examine the growth of hypocotyls, seeds were placed on agar plates and illuminated with 45–55 μmol m^-2^ s^-1^ white light for 24 h. The plates of seeds were then grown vertically for 6 days under continuous red or far-red light. At that time, the plates were scanned with a flat-bed scanner and seedling length measured using ImageJ (ver. 1.43u).

### RNA ISOLATION AND QUANTITATIVE REAL-TIME REVERSE TRANSCRIPTASE (qRT)-PCR

The transcript abundance of several *Arabidopsis* genes that encode GA and ABA metabolic enzymes was examined using quantitative real-time reverse transcriptase (qRT)-PCR. This included gene transcripts for *GA3 oxidase 1* and *2* (*GA3ox1*, *GA3ox2*) encoding for two GA biosynthesis enzymes, *GA2ox2* encoding for a GA degradation enzyme, *zeaxanthin epoxidase* (*ZEP*), *9-cis-epoxycarotenoid dioxygenase6* and *9* (*NCED6*, *NCED9*) encoding for three ABA biosynthesis enzymes, and ABA* 8*′*-hydroxylase* (*CYP707A2*) encoding for an ABA degradation enzyme. For this, total RNA was isolated from either 25 mg dry seeds or 25 mg (dry weight) of seeds that were imbibed in half strength MS and light treated as specified for the indicated times. RNA was isolated according to methods modified from Meng and Feldman (2010). Instead of resuspending the pellet in Trizol, the RNA was further purified using the Spectrum Plant Total RNA Kit (Sigma, St. Louis, MO, USA). Total RNA was treated with DNase I (Invitrogen, Carlsbad, CA, USA) and 800 ng of the RNA was used for cDNA synthesis with the ImProm-II Reverse Transcription System (Promega, Madison, WI, USA) according to the manufacturer’s instructions. Each qPCR reaction consisted of 5 μL of SsoFast EvaGreen Supermix (Bio-Rad, Hercules, CA, USA), 0.5 μL each of the forward and Reverse primers (10 μM) and 4 μL of cDNA diluted 1:8. The qPCR reactions were run on a Bio-Rad iQ5 Real-Time PCR Detection System (Bio-Rad, Hercules, CA, USA) with the following conditions: an initial denaturation step of 95^∘^C for 1 min followed by 45 cycles of 15 s at 95°C, 30 s at 58°C, and 10 s at 72°C.

Transcript data were normalized to At3g12210 (Dekkers et al., 2011) using the method of Livak and Schmittgen (2001) for each seed line for each condition to obtain the relative amounts of target gene transcripts between plant backgrounds for each treatment. The primers used for *GA3ox2* were 5′-GTTCTTTAATAAGAAGATGTGGTCCG-3′ (forward) and 5′CATCAACTTGGCTGCCAACTTT-3′ (reverse). Other primers used have been previously described (Seo et al., 2004; Kim et al., 2012; Shu et al., 2013).

### STATISTICS

Data were analyzed with Student’s *t-*tests and considered statistically different with a *P* value <0.05.

## RESULTS

### ETR1 INHIBITS SEED GERMINATION AFTER EXPOSURE TO FAR-RED LIGHT

**FIGURE 1:**
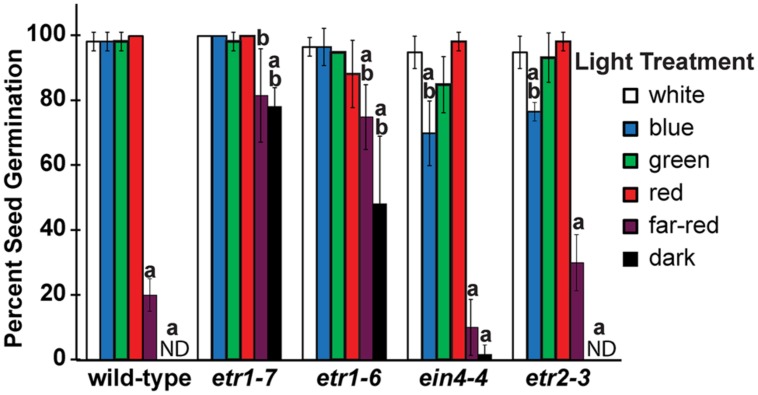
**Ethylene receptors affect germination in response to different wavelengths of light.** Seed germination of wild-type seeds was compared to ethylene receptor loss-of-function mutants. Seeds were treated with 4 h of white light, followed by 3 h of different colors of monochromatic light or white light as indicated. For dark controls, seeds were treated for 5 min with far-red light immediately after sowing without a pre-treatment with white light. The seeds were then placed in darkness for 7 days. At that time seed germination was measured and the percent of seed germination calculated. Plots show the average ± SD from at least three biological replicates. ND denotes no germination detected. Data were analyzed by *t*-tests and differences considered statistically significant with *P* < 0.05. ^a^Significant difference from white light for that ecotype; ^b^Significant difference of mutant from wild-type after the same light treatment.

We recently reported that loss of ETR1 led to better seed germination during salt stress when seeds were germinated in white light (Wilson et al., 2014). During the course of those experiments we noted that loss of ETR1 also caused better seed germination during salt treatment when the seeds were germinated in darkness (data not shown). This led us to determine the role of individual ethylene receptor isoforms on seed germination under different light conditions. To do this, we measured the percent of seed germination for receptor loss-of-function mutants 7 days following a 3 h treatment with white, blue, green, red, or far-red light as described in Section “Materials and Methods.” Following white light treatment, all seed lines reached at least 95% germination (**Figure 1**). The extent of wild-type seed germination was not altered by blue, green, or red light. However, germination of wild-type seeds was drastically reduced by treatment with far-red light. This is consistent with prior research (Shinomura et al., 1994). By contrast, the effect of far-red light on the germination of *etr1-6* and *etr1-7* loss-of-function mutants was significantly reduced even though responses to blue, green, and red light were not altered by the loss of ETR1. This effect of *etr1* mutants was observed with multiple seed batches. Loss of ETR2 or EIN4 did not affect responses to far-red light, but did have a small and statistically significant effect on germination in blue light (**Figure 1**). Loss of ERS1 or ERS2 had no measurable effect on germination in any of the light conditions tested (data not shown). Interestingly, when germination occurred in darkness without a white light pre-treatment, wild-type, *etr2-3*, and *ein4-4* seeds failed to germinate or germinated very poorly, whereas *etr1* loss-of-function mutants germinated to an extent that was similar to germination after far-red illumination (**Figure 1**). These data suggest that ETR2 and EIN4 promote seed germination after exposure to blue light and ETR1 inhibits germination after exposure to far-red light and in darkness.

### ETR1 FUNCTIONS OPPOSITELY TO ETR2 TO INFLUENCE SEED GERMINATION AFTER EXPOSURE TO FAR-RED LIGHT

**FIGURE 2:**
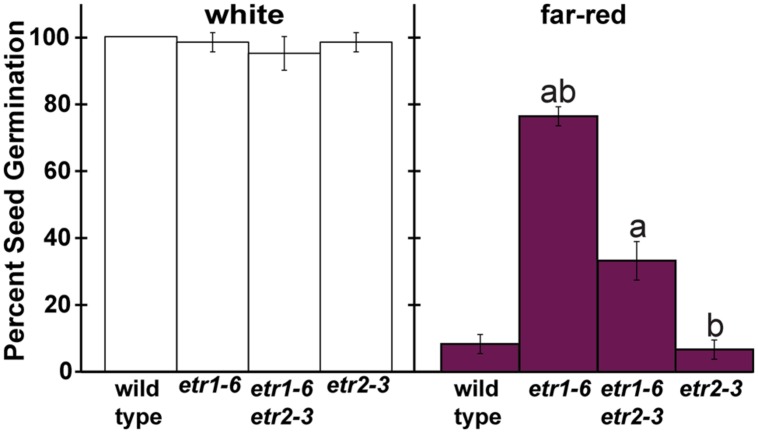
**ETR1 functions oppositely to ETR2 to control seed germination after treatment with far-red light.** The percent of wild-type, *etr1-6*,* etr2-3*, and *etr1-6;etr2-3* seed germination was determined 7 days after a far-red light treatment. For comparison, germination after a white light treatment is included. Plots show the average ± SD from at least three biological replicates. Data were analyzed by *t*-tests and differences considered statistically significant with *P* < 0.05.^a^Statistical difference from wild-type in that light condition; ^b^Significant difference of the single mutant from the double.

In seed germination under salt stress, *etr2-3* loss-of-function mutants were shown to have reduced germination compared to wild-type seeds and ETR1 and ETR2 were found to act additively (Wilson et al., 2014). In contrast, under the far-red light conditions used, the germination of *etr2-3* mutants was indistinguishable from wild-type seeds (**Figure 1**). However, an effect of ETR2 on seed germination in these conditions might have been masked by the fact that the wild-type seeds germinated poorly and a further reduction might have been obscured. To determine whether or not ETR1 and ETR2 act additively to control seed germination after far-red light, we compared seed germination of *etr1-6* and *etr2-3* to germination of *etr1-6;etr2-3* double mutants. Following a 3 h white light treatment, the seed germination of the single and double mutants were not significantly different and reached at least 95% germination (**Figure 2**). A different pattern emerged after a 3 h far-red light treatment. Here, the *etr1-6* seeds germinated significantly better and the *etr2-3* seeds germinated significantly worse than the *etr1-6;etr2-3* double mutants. These results show that ETR1 and ETR2 have opposite effects on seed germination after far-red light treatment.

### THE ETR1 RECEIVER DOMAIN IS NOT REQUIRED FOR ETR1 FUNCTION IN SEED GERMINATION AFTER EXPOSURE TO FAR-RED LIGHT

**FIGURE 3:**
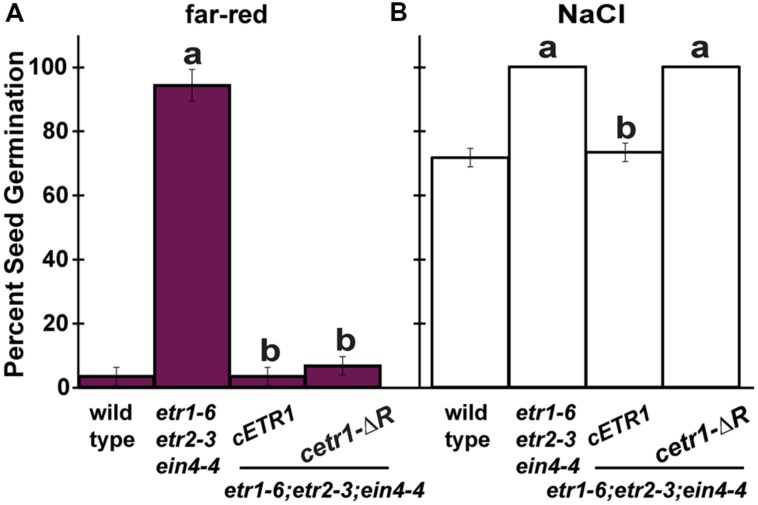
**After far-red illumination, the better germination of *etr1;etr2;ein4* triple mutants is reversed by both a full-length and truncated ETR1 transgene lacking the receiver domain. (A)** The percent of germination 7 days after treatment with far-red light for wild-type, *etr1-6;etr2-3;ein4-4* triple mutants and these triple mutants transformed with cDNA for full-length *ETR1* (*cETR1*) or a truncated *ETR1* lacking the receiver domain (*cetr1-ΔR*) was determined. **(B)** For comparison, the percent germination of these seed lines 7 days after sowing on 150 mM NaCl in the presence of continuous white light was determined. Data were analyzed by *t*-tests and differences considered statistically significant with *P* < 0.05. ^a^Significant difference from wild-type germinated under the same conditions.^b^Significant rescue of germination by the transgene.

We previously showed that *etr1-6;etr2-3;ein4-4* triple mutants germinate better than wild-type seeds during salt stress under white light (Wilson et al., 2014). Interestingly, transformation of this triple mutant with a cDNA construct for full-length *ETR1* (*cETR1*) resulted in a reduction in germination, whereas transformation with a truncated *ETR1* transgene lacking the receiver domain (*cetr1-ΔR*) failed to alter germination (Wilson et al., 2014). Both constructs are under the control of the *ETR1* promoter and have been shown to be expressed and functional (Kim et al., 2011) supporting the idea that the full-length ETR1 receptor is required to affect seed germination during salt stress under white light. To determine whether or not the ETR1 receiver domain is also required to modulate seed germination after treatment with far-red light, we examined the seed germination of these transformants. Comparable to our results with germination on salt, the *etr1-6;etr2-3;ein4-4* triple mutants germinated better than wild-type seeds after illumination with far-red light (**Figure 3**). However, in contrast to germination on salt, both the full-length and truncated *ETR1* transgenes reduced seed germination to wild-type levels after far-red illumination indicating that the receiver domain of ETR1 is not required to function in this trait. When transferred to white light conditions for 7 days, all seed lines reached at least 98% germination (data not shown). This pattern of rescue is different from what we obtained when studying the role of ETR1 in the control of germination during salt stress in white light (Wilson et al., 2014). To confirm this difference, we germinated seeds in white light under salt stress. Under salt stress, wild-type seeds reached approximately 72% germination while the *etr1-6;etr2-3;ein4-4* triple mutants reached 100% germination (**Figure 3**). The full-length *cETR1*, but not the truncated *cetr1-ΔR* transgene, reduced germination to wild-type levels as previously reported (Wilson et al., 2014). These results reveal that the function of ETR1 varies depending upon environmental conditions.

### ETHYLENE MAY HAVE A ROLE IN MEDIATING THE EFFECTS OF ETR1 ON GERMINATION AFTER EXPOSURE TO FAR-RED ILLUMINATION

**FIGURE 4:**
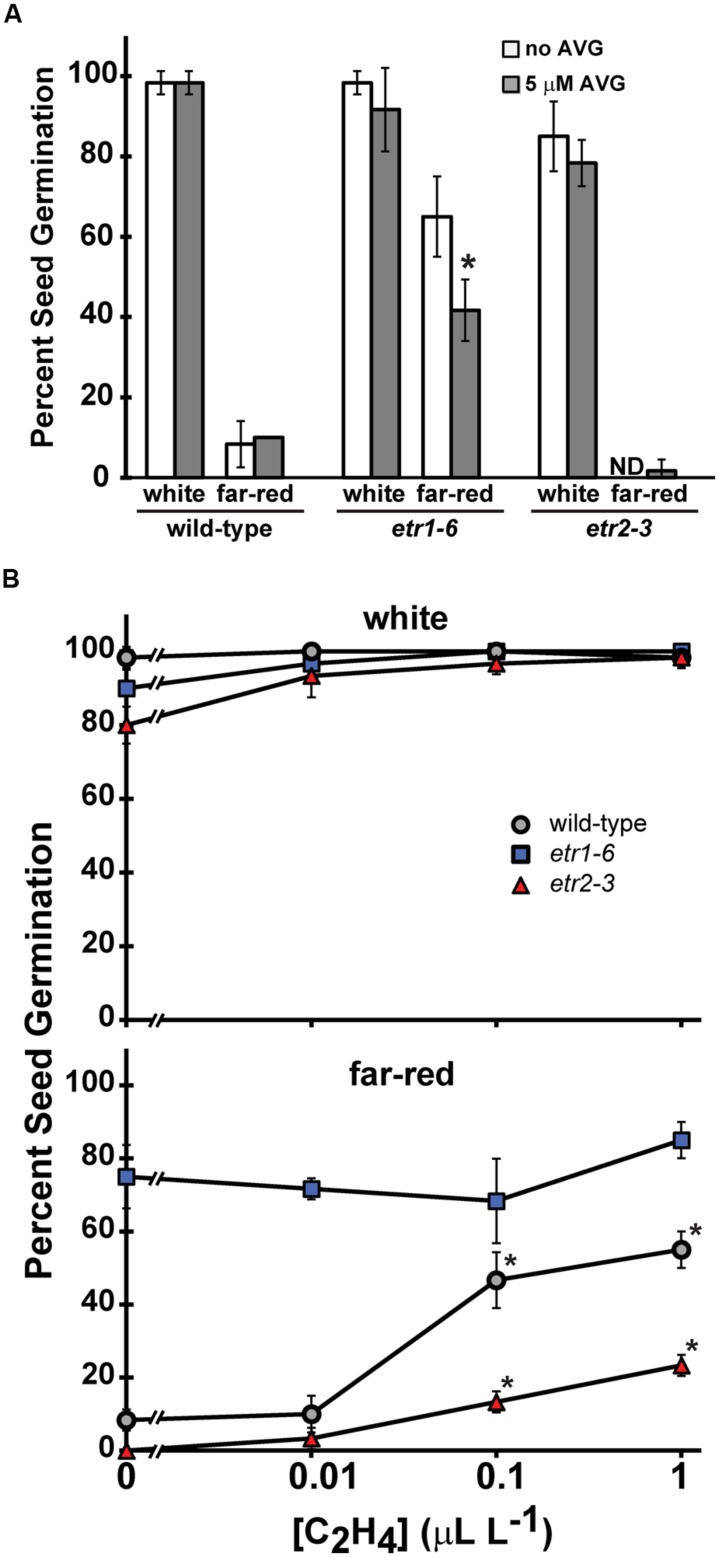
**The effects of L-α-(2-amino ethoxyvinyl)-glycine (AVG) and ethylene on seed germination after treatment with far-red light.** The number of seeds that germinated after far-red or white illumination was counted and the percent of seeds that germinated was calculated. **(A)** Seeds on plates containing the ethylene biosynthesis inhibitor, AVG, were treated with far-red light and then germinated for 7 days in the dark. The average ± SD from at least three biological replicates is plotted. ND denotes no germination detected. Data were analyzed by *t*-tests and differences considered statistically significant with *P* < 0.05. *Denotes that AVG had a significant effect compared to the non-treated control in that light condition. **(B)** The effects of increasing concentrations of ethylene as indicated were measured after treatment with white light or far-red light. The average ± SD from at least three biological replicates is plotted. Data were analyzed by *t*-tests and differences considered statistically significant with *P* < 0.05. *Denotes that ethylene significantly increased the percent of germination after-far red light over the ethylene untreated control.

In order to determine whether differences in ethylene production or sensitivity are responsible for the difference between the *etr1* loss-of-function mutants and wild-type in germination following far-red treatment, we examined the effect of ethylene and the ethylene biosynthesis inhibitor, AVG, on germination. Following white light treatment, AVG did not have a statistically significant effect on the germination of wild-type, *etr1-6* or *etr2-3* seeds (**Figure 4A**). AVG also did not affect wild-type or *etr2-3* germination following far-red treatment. However, germination of *etr1-6* was statistically reduced by 20% by AVG after far-red light (**Figure 4A**). Although AVG reduced germination of *etr1-6* seeds in far-red, this mutant still had better seed germination than wild-type. These observations suggest that part of the difference in germination between wild-type and the *etr1-6* mutants may be due to alterations in ethylene levels. To more directly determine this, we measured ethylene levels of germinating seeds after white or far-red light treatment. We found that ethylene levels were indistinguishable between *etr1-6* and wild-type seeds during germination in both conditions (data not shown) suggesting that higher ethylene levels are not responsible for the enhanced germination of *etr1-6* seeds after far-red illumination.

Ethylene can stimulate seed germination of lettuce and cocklebur after far-red illumination (Abeles and Lonski, 1969; Burdett and Vidaver, 1971; Esashi et al., 1987). We wished to determine if this was the case for *Arabidopsis* too and to see if the *etr1-6* and *etr2-3* mutants had altered ethylene sensitivity when assayed in these conditions. One explanation for *etr1* loss-of-function mutants germinating better than wild-type is that they are more sensitive to ethylene. This has been observed when assaying growth of dark-grown *Arabidopsis* seedlings (Hua and Meyerowitz, 1998; Cancel and Larsen, 2002), but is not true for seeds germinating on salt (Wilson et al., 2014). After white light, 0.01 ppm ethylene increased the germination of *etr2-3* seeds slightly, but significantly (*P* < 0.05), and higher concentrations of ethylene caused no measurable additional increase in germination. By contrast, ethylene had no measurable effect on the germination of wild-type or *etr1-6* seeds after white light illumination (**Figure 4B**). After treatment with far-red light, ethylene increased seed germination of wild-type seeds (**Figure 4B**). By contrast, ethylene had no measurable effect on germination of the *etr1*-*6* mutants indicating that these mutants are largely un-responsive to ethylene for this trait. We also examined *etr2-3* mutants and found that ethylene enhanced germination of these seeds after far-red illumination, but the effect was smaller than seen with wild-type seeds. The highest concentration of ethylene used (1 ppm) failed to completely eliminate the differences in germination between *etr1-6*, *etr2-3*, and wild-type seeds. This is at least 1000-fold higher than the concentration of ethylene released by the germinating seeds under these conditions. Thus, ethylene may have a role in mediating the differences in germination between wild-type and *etr1* mutants, but other factors are likely to be more important.

### ABA AND GA HAVE A ROLE IN MEDIATING THE EFFECTS OF ETR1 ON GERMINATION AFTER EXPOSURE TO FAR-RED ILLUMINATION

There are complex interactions between hormones and light to control germination (reviewed by Finkelstein et al., 2008; Lau and Deng, 2010; Leivar and Quail, 2010). We previously found that ETR1 predominantly affects ABA sensitivity to alter seed germination during salt stress (Wilson et al., 2014). We therefore were curious to know if ETR1 is also predominantly affecting seed germination after treatment with far-red light in the same way.

**FIGURE 5:**
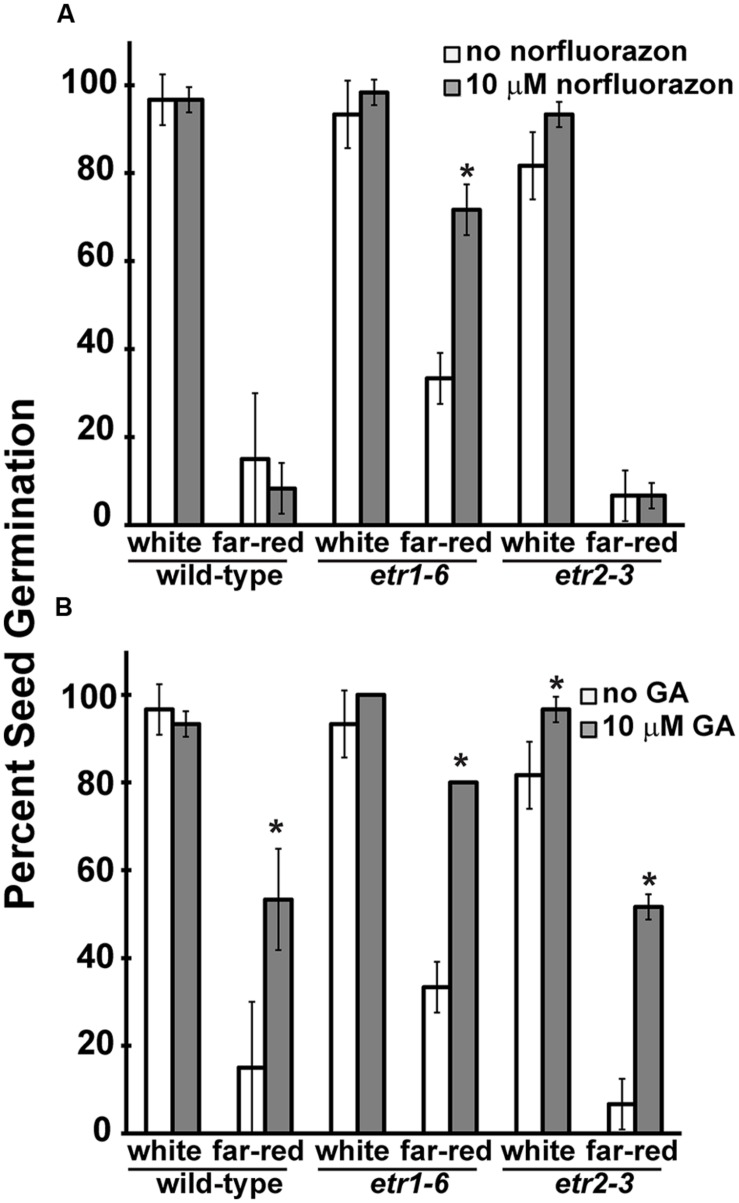
**The effects of gibberellic acid (GA) and norflurazon on seed germination after treatment with far-red light.** Wild-type, *etr1-6*, and *etr2-3* seeds were germinated after treatment with white or far-red light. Seed germination was then measured 7 days later. The effects of **(A)** norflurazon and **(B)** GA were assessed. All experiments were performed in triplicate. The average percent seed germination ± SD is plotted. Data were analyzed by *t*-tests and differences considered statistically significant with *P* < 0.05. *Denotes that the compound had a significant effect compared to the non-treated control in that light condition.

Abscisic acid is well known for inhibiting germination and GA for stimulating *Arabidopsis* seed germination (Hilhorst and Karssen, 1992; Garciarrubio et al., 1997). In order to test whether differences in ABA or GA or both were responsible for the differences in germination following far-red light treatment, we treated the seeds with an ABA biosynthesis inhibitor, norflurazon, or with GA. Initially, these chemicals were dissolved in ethanol to yield a final concentration of 0.1% (v/v) ethanol. However, this concentration of ethanol significantly (*P* < 0.05) reduced germination of *etr1-6* and *etr2-3* seeds after white light treatment to 55 ± 22 and 45 ± 9%, respectively. As previously observed, this concentration of ethanol had no measureable effect on the germination of Columbia seeds (Hirayama et al., 2004). Even though norflurazon and GA affected germination as expected, this added effect of the ethanol made interpretation of the data difficult. Because of this, we used stocks that yielded a final ethanol concentration of 0.01% (v/v). At this concentration of ethanol, there was no statistical difference (*P* < 0.05) between wild-type and the mutants following white light treatment (**Figure 5**). However, following far-red treatment, this concentration of ethanol significantly (*P* < 0.05) reduced the germination of *etr1-6* (**Figure 1** vs. **Figure 5**). In **Figure 1**, that has no added ethanol, *etr1-6* reached 75 ± 10% germination, but when ethanol was added to the plates (**Figure 5**) *etr1-6* only reached 33 ± 6% germination following far-red treatment. These experiments were conducted at the same time and with the same seed batches. We found that addition of 10 μM norflurazon, resulted in a significant improvement of *etr1-6* germination following far-red treatment, but surprisingly had no effect on wild-type or *etr2-3* seed germination (**Figure 5A**). Treatment with 10 μM GA significantly increased the germination following far-red treatment of all three seed lines tested (*P* < 0.05), but was not able to improve the germination of wild-type and *etr2-3* to that seen by *etr1-6* (**Figure 5B**). These data suggest that ETR1 is having a complex effect on ABA and GA to control seed germination after far-red illumination. This is somewhat different from the role of ETR1 in controlling seed germination during salt stress (Wilson et al., 2014).

**FIGURE 6:**
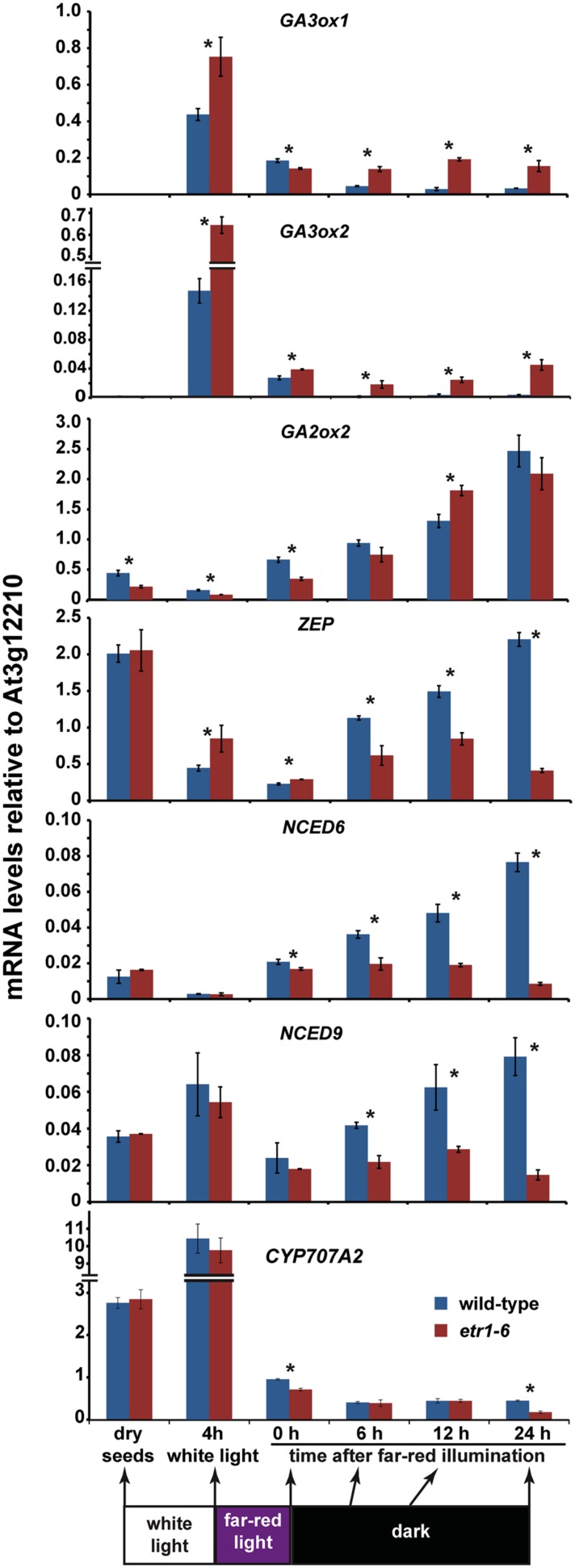
**Transcript levels for select GA and ABA metabolism genes are affected by loss of ETR1.** The levels of transcript for *GA3ox1*, *GA3ox2*, *GA2ox2*, *ZEP*, *NCED6*, *NCED9*, and *CYP707A2* in wild-type and *etr1-6* mutants at various times during germination were measured using qRT-PCR. Data were normalized to the levels of At3g12210 in each seed line at each stage of germination. Transcript levels of dry seeds and imbibed seeds treated with light as indicated were compared. Data were analyzed by *t*-tests and differences considered statistically significant with *P* < 0.05. *Denotes that the levels of transcript at that time-point are statistically different between wild-type and *etr1-6* seeds.

To explore this complex interaction more, we used qRT-PCR to examine the time-course of changes in transcript levels of selected genes during germination under the conditions used above. We chose genes encoding for enzymes for GA biosynthesis (*GA3ox1*, *GA3ox2*), GA degradation (*GA2ox2*), ABA biosynthesis (*ZEP*, *NCED6*, *NCED9*), and ABA degradation (*CYP707A2*). These genes are important for seed development and germination (Yamauchi et al., 2004, 2007; Lefebvre et al., 2006; Okamoto et al., 2006). Generally consistent with prior studies (Yamauchi et al., 2004; Seo et al., 2006; Finch-Savage et al., 2007), imbibing wild-type seeds in white light led to large increases in the transcript abundance of *GA3ox1*, *GA3ox2*, and *CYP707A2* and significant (*P* < 0.05) decreases in *GA2ox2*, *ZEP*, and *NCED6* (**Figure 6**). The transcript abundance of *NCED9* increased slightly, but overall these results are predicted to lead to increased GA levels and decreased ABA levels. Immediately following treatment with far-red light, the transcript abundance of *GA3ox1*, *GA3ox2*, *ZEP*, *NCED9*, and *CYP707A2* decreased and the levels of *GA2ox2* and *NCED6* increased in wild-type seeds. Overall, these transcriptional changes are predicted to cause ABA levels to rise and GA levels to decrease compared to white light and lead to inhibited germination. In the wild-type seeds, the transcript levels of each gene showed different patterns of change over the 24 h period following far-red illumination. *GA3ox1* and *GA3ox2* showed a small decrease 6 h following far-red light but then plateaued at a very low level and *CYP707A2* showed very little change in transcript abundance after far-red illumination. By contrast, the transcript levels of *ZEP*, *NCED6*, and *NCED9* increased with time after far-red light treatment.

The effect of the *etr1-6* mutation on the transcript levels of these genes was evident at various times during germination. After white-light treatment, the *etr1-6* mutants had higher levels of *GA3ox1*, *GA3ox2*, and *ZEP* and slightly lower levels of *GA2ox2* (*P* < 0.05) compared to the wild-type seeds (**Figure 6**). Immediately following treatment with far-red light, the levels of *GA3ox1*, *GA2ox2*, *NCED6*, and *CYP707A2* were slightly but significantly (*P* < 0.05) lower in *etr1-6* mutants compared to wild-type and *GA3ox2* and *ZEP* were slightly, but significantly higher in the mutants compared to wild-type. More differences between the *etr1-6* seeds and wild-type became apparent with increasing time after treatment with far-red light with a trend toward higher levels of *GA3ox1* and *GA3ox2* and lower levels of *ZEP*, *NCED6*, and *NCED9* in the *etr1-6* mutants compared to wild-type. The differences in the levels of *ZEP*, *NCED6*, and *NCED9* appear to arise because the levels of these gene transcripts increased in the wild-type seeds, but remained fairly constant in the *etr1-6* mutants. Since the transcript levels of GA and ABA metabolic genes generally correlate with the levels of GA and ABA, respectively (Qin and Zeevaart, 1999; Iuchi et al., 2001; Reid et al., 2002; Seo et al., 2004; Lefebvre et al., 2006; Reinecke et al., 2013), our results suggest that the *etr1-6* seeds are likely to have higher levels of GA and lower levels of ABA than wild-type seeds following treatment with far-red light. These alterations in GA and ABA levels are predicted to cause better germination of the mutant seeds compared to the wild-type seeds after far-red illumination.

### *ETR1* GENETICALLY INTERACTS WITH *PHYA* AND *PHYB* TO CONTROL GERMINATION

**FIGURE 7:**
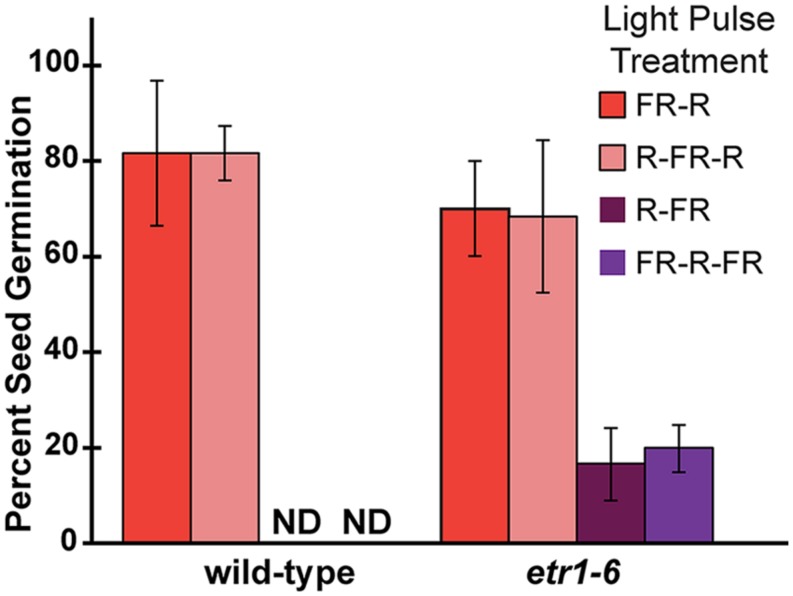
**The effect of ETR1 on germination after far-red light treatment is reversed by red light.** Seeds were treated with white light for 3 h, followed by a series of 5 min pulses of red (R) and far-red (FR) light as shown. The percent of seeds that germinated was determined 7 days later. The average ± SD from at least three biological replicates is plotted. ND denotes no germination detected.

The phy have an important role in seed germination and responses to far-red light are mediated by the phy (Shinomura et al., 1994, 1996; Poppe and Schäfer, 1997; Hennig et al., 2002; Rösler et al., 2007) suggesting that ETR1 is affecting events influenced by the phy. The phy act as photoreversible switches driven by red and far-red light to control germination of seeds including germination of *Arabidopsis* (Shinomura et al., 1994; Casal and Sánchez, 1998). To confirm that loss of ETR1 is affecting phy-influenced germination, we treated seedlings with a series of 5 min pulses of red and far-red light followed by darkness (**Figure 7**). From this we found that there was no measurable difference in wild-type and *etr1-6* germination rates in seeds treated with a pulse series that ended with red light. By contrast, if the pulse series ended with far-red light, wild-type seedlings failed to germinate, but approximately 20% of the *etr1-6* seeds germinated.

**FIGURE 8:**
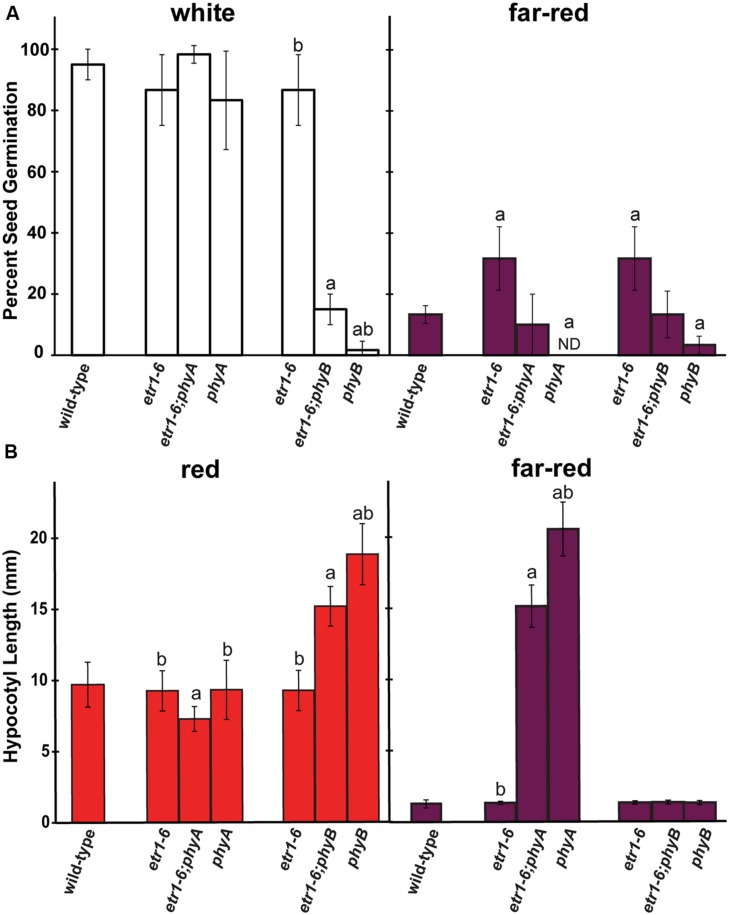
**Epistasis analysis of *ETR1* and the phytochromes (phy), *PHYA* and *PHYB* after light treatment. (A)** Seeds were treated with white light for 3 h, followed by either 4 h of white or far-red light as shown. The percent of seed germination was then determined 7 days later. The average ± SD from at least three biological replicates is plotted. ND denotes no germination detected. **(B)** Seeds were treated with white light for 24 h followed by continuous illumination with red or far-red light for 6 days as shown. The lengths of the hypocotyls were then measured. The average ± SD is plotted. In both panels, the data were analyzed by *t*-tests and differences considered statistically significant with *P* < 0.05. ^a^Statistical difference from wild-type in that light condition; ^b^Significant difference of the single mutant from the double.

Of the phy, phyA and phyB have the predominant roles in controlling *Arabidopsis* seed germination (Shinomura et al., 1994, 1996; Rösler et al., 2007). To further explore the interaction between ETR1 and the phy, we generated *etr1-6;phya-t* and *etr1-6;phyb-9* double mutants. After treatment with white light, the germination rates of *etr1-6*, *etr1-6;phya-t*, and *phya-t* were indistinguishable from wild-type seeds (**Figure 8A**). By contrast, the *phyb-9* seeds had severely diminished germination after white light treatment, consistent with previous reports (Reed et al., 1994; Shinomura et al., 1994). The *etr1-6;phyb-9* double mutants had a percent germination worse than the *etr1-6* single mutants and slightly better than the *phyb-9* mutants following white light treatment. After treatment with far-red light, the *phya-t* and *phyb-9* mutants had significantly worse germination than wild-type seeds and the *etr1-6* mutants significantly better germination (**Figure 8A**). Both the *etr1-6;phya-t* and *etr1-6;phyb-9* double mutants had a percent germination intermediate between the single mutants. However, these were not statistically different.

We also examined hypocotyl growth in red and far-red light (**Figure 8B**). Consistent with previous studies, the *phyB* mutants grew taller than wild-type in red light and the *phyA* mutants grew taller than wild-type in far-red light (Reed et al., 1994; Quail et al., 1995; Smith et al., 1997). The growth of the *etr1-6* seedlings was similar to wild-type in both light conditions. In red light, the *etr1-6;phyb-9* double mutants were shorter than the *phyb-9* mutants and taller than the *etr1-6* mutants. Similarly, in far-red light the *etr1-6;phya-t* double mutants were shorter than the *phya-t* seedlings and taller than the *etr1-6* seedlings. The *etr1-6;phya-t* double mutants were also slightly, but significantly, shorter than either single mutant in red light. Together, these results on seed germination and hypocotyl growth show that *ETR1* genetically interacts with *PHYA* and *PHYB*.

### ETR1 AFFECTS GERMINATION INDEPENDENTLY OF LIGHT

**FIGURE 9:**
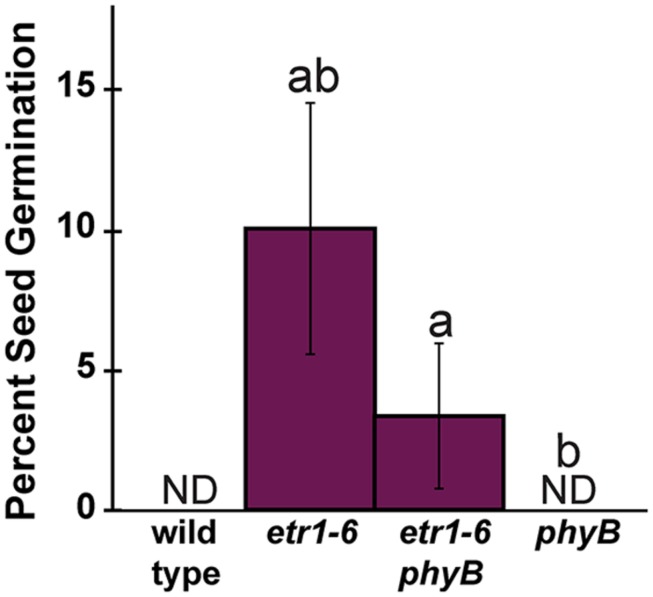
**Epistasis analysis of *ETR1* and *PHYB* in the dark.** Seeds were germinated in the dark for 7 days and the percent of seed germination was then determined. The average ± SD from at least three biological replicates is plotted. ND denotes no germination detected. The data were analyzed by *t*-tests and differences considered statistically significant with *P* < 0.05. ^a^Statistical difference from wild-type in that light condition; ^b^Significant difference of the single mutant from the double mutant.

From the above experiments, it is unclear whether or not ETR1 is affecting responses to light by affecting signaling from phyA or phyB. We have previously shown that *etr1-6* loss-of-function mutants are more sensitive to ABA when measuring seed germination under white light (Wilson et al., 2014) indicating that the effects of ETR1 could be independent of these photoreceptors. Since phyB has the major role in controlling germination in the dark (Bentsink and Koorneef, 2008), we examined the effect of ETR1 on the germination of *phyb-9* mutants in the dark. In dark conditions, wild-type and *phyb-9* seeds failed to germinate, whereas *etr1-6* mutants did germinate (**Figure 9**). The *etr1-6;phyb-9* also germinated under these conditions but had a percent germination lower than the *etr1-6* single mutant seeds. This indicates that ETR1 can affect germination independently of light.

## DISCUSSION

It has been suggested that there is a network of two-component receptors in plants that fine-tune signaling (Hwang et al., 2002; Hass et al., 2004; Wilson et al., 2014). In this study, we found that ETR1, a two-component-like receptor for ethylene, inhibits germination after far-red light treatment and in the dark. This is similar to results we obtained when examining germination during salt stress (Wilson et al., 2014). However, in the prior study, the effect of ETR1 was to mainly affect ABA signaling or synthesis. In the current study, ETR1 was found to affect ABA, GA, and perhaps ethylene. It has previously been shown that in *Arabidopsis* phyA and B affect the transcript abundance of *ETR2* and *ERS1* (Tepperman et al., 2004), and phy interacting factor1 (PIF1), which is important for phy signaling, affects the transcript abundance of *ETR2* and *EIN4* (Oh et al., 2009). Additionally, phyB is involved in the circadian control of ethylene synthesis in sorghum (Finlayson et al., 1998) showing that there is likely to be signaling cross-talk from the phy to ethylene signaling and synthesis. Results presented in the current study show that there is also signaling from ETR1, but not the other four ethylene receptor isoforms, that affects responses to red and far-red light.

Previous research has shown that the receiver domain of ETR1 affects receptor function in the control of growth of dark-grown seedlings (Binder et al., 2004, 2006; Qu and Schaller, 2004; Kim et al., 2011) and germination during salt stress under white light (Wilson et al., 2014). In the current study, we found that transformation of *etr1-6;etr2-3;ein4-4* triple mutants with either *cETR1* or *cetr1-ΔR* rescued germination after far-red light treatment to wild-type levels showing that the receiver domain of ETR1 is not required in the control of this trait. This is intriguing because the receiver domain is required for ETR1 function in the control of germination during salt stress under white light (Wilson et al., 2014; this study). These results indicate that environmental conditions affect the function of the ETR1 ethylene receptor to regulate seed germination. The mechanism for this is not known but one possibility is that a downstream target unique to ETR1 is altered upon illumination. There is precedence for this since light regulates the levels of one downstream target of EIN3, PIF3, to alter the effects of ethylene on plant growth in the dark versus light (Zhong et al., 2012).

A major question is whether or not the effect of ETR1 on seed germination is ethylene-dependent or not. We found that the ethylene biosynthesis inhibitor, AVG, partially reversed the effect of *etr1-6* on germination after treatment far-red light, but addition of ethylene up to 1 ppm had no effect on germination of the mutant after far-red illumination. It is possible that the *etr1* loss-of-function mutants are producing high levels of ethylene and are saturated for ethylene responses. However, this seems unlikely since *etr1-6* produced very low levels of ethylene that were indistinguishable from the germinating wild-type seeds after illumination with far-red light. Transfer of these seeds to white light conditions resulted in an increase in germination showing that the seeds were still capable of better germination. Additionally, AVG has effects that are independent of its effect on ethylene biosynthesis (Larsen and Chang, 2001; Soeno et al., 2010) confounding the results with this compound. Thus, it is seems unlikely that ethylene is the major factor contributing to the differences in germination between wild-type and *etr1* loss-of-function mutants observed in this study.

Results presented here indicate that ETR1 is affecting germination by affecting the levels of GA and ABA. We observed that the transcript abundance of genes encoding certain GA and ABA metabolic genes are altered in *etr1-6* mutants consistent with higher GA levels and lower ABA levels after far-red light treatment in the *etr1* loss-of-function mutants compared to wild-type. Additionally, we have previously shown that *etr1-6* mutants are less sensitive to ABA than wild-type (Wilson et al., 2014). Together, these results would explain the better germination of the *etr1* loss-of-function mutants. Consistent with these results, application of GA improved the germination of all seed lines and norflurazon improved seed germination of *etr1-6* mutants.

Another major question is whether ETR1 is affecting germination via the phy signaling pathway, via a phy-independent pathway, or both. Previous studies provide a tentative link between ETR1 and phyB (Urao et al., 2000; Sweere et al., 2001; To et al., 2004; Salomé et al., 2006; Scharein et al., 2008). However, our data suggest that ETR1 is affecting responses to light that are independent of the phy. Support for this is our observation that *etr1* loss-of-function mutants are more sensitive to ABA in white light (Wilson et al., 2014) and germinate better than wild-type seeds in the dark (this study). Additionally, *etr1;phyb* double mutants germinated in the dark, whereas *phyb* single mutants did not. Similarly, after treatment with far-red light the *phyA* mutants did not germinate but the *etr1;phyA* double mutants did germinate. Thus, the effects of ETR1 on germination are occurring in the absence of actively signaling phy. These observations coupled with our observation that ETR1 affects the transcript levels of genes encoding for ABA and GA metabolic enzymes support a model where ETR1 is affecting germination by altering ABA and GA levels and ABA sensitivity in parallel with the effects of phy signaling on these parameters.

## Conflict of Interest Statement

The authors declare that the research was conducted in the absence of any commercial or financial relationships that could be construed as a potential conflict of interest.
